# The importance of assessing self-reported HIV status in bio-behavioural surveys

**DOI:** 10.2471/BLT.15.162933

**Published:** 2016-04-25

**Authors:** Lisa G Johnston, Miriam Lewis Sabin, Dimitri Prybylski, Keith Sabin, Willi McFarland, Stefan Baral, Andrea A Kim, H Fisher Raymond

**Affiliations:** a Global Health Sciences, University of California - San Francisco, San Francisco, United States of America (USA).; b Global Fund to Fight AIDS, Tuberculosis and Malaria, Geneva, Switzerland.; c Global AIDS Program Asia Regional Office, Centers for Disease Control and Prevention, Nonthaburi, Thailand.; d Joint United Nations Programme on HIV/AIDS, 20 avenue Appia, CH-1211 Geneva 27, Switzerland.; e Center for Public Health and Human Rights, Johns Hopkins Bloomberg School of Public Health, Baltimore, USA.; f Division of Global HIV/AIDS, United States Centers for Disease Control and Prevention, Atlanta, USA.

## Abstract

In bio-behavioural surveys measuring prevalence of infection with human immunodeficiency virus (HIV), respondents should be asked the results of their last HIV test. However, many government authorities, nongovernmental organizations, researchers and other civil society stakeholders have stated that respondents involved in such surveys should not be asked to self-report their HIV status. The reasons offered for not asking respondents to report their status are that responses may be inaccurate and that asking about HIV status may violate the respondents’ human rights and exacerbate stigma and discrimination. Nevertheless, we contend that, in the antiretroviral therapy era, asking respondents in bio-behavioural surveys to self-report their HIV status is essential for measuring and improving access to – and coverage of – services for the care, treatment and prevention of HIV infection. It is also important for estimating the true size of the unmet needs in addressing the HIV epidemic and for interpreting the behaviours associated with the acquisition and transmission of HIV infection correctly. The data available indicate that most participants in health-related surveys are willing to respond to a question about HIV status – as one of possibly several sensitive questions about sexual and drug use behaviours. Ultimately, normalizing the self-reporting of HIV status could help the global community move from an era of so-called exceptionalism to one of destigmatization – and so improve the epidemic response worldwide.

## Résumé

Dans les enquêtes bio-comportementales évaluant la prévalence de l'infection par le virus de l'immunodéficience humaine (VIH), il conviendrait de demander aux répondants le résultat de leur dernier test de dépistage du VIH. Mais nombre d'autorités gouvernementales, d'organisations non gouvernementales, de chercheurs et d'acteurs de la société civile sont défavorables à ce que l’on demande aux répondants de déclarer leur statut VIH dans ce type d'enquêtes. Parmi les raisons invoquées pour ne pas demander le statut VIH figure le fait que les réponses peuvent être inexactes et qu'une telle demande pourrait transgresser les droits humains des répondants et accentuer les problèmes de stigmatisation et de discrimination. Néanmoins, nous soutenons qu'aujourd'hui, à l'ère de la thérapie antirétrovirale, le fait de demander leur statut VIH aux répondants des enquêtes bio-comportementales est essentiel à des fins d'évaluation des services de soins, traitement et prévention de l'infection à VIH et pour en optimiser l'accès et la couverture. Cela est également fondamental pour évaluer la véritable ampleur des besoins non satisfaits dans la gestion de l'épidémie de VIH et pour correctement interpréter les comportements associés à l'acquisition et à la transmission du VIH. Les données disponibles indiquent que la plupart des participants aux enquêtes liées à la santé acceptent de répondre à une question sur leur statut VIH, parmi d'autres questions sensibles concernant les comportements sexuels et l'usage de drogues. Enfin, une normalisation de l'auto-déclaration du statut VIH pourrait aider la communauté mondiale à dépasser ce que l'on appelle « l'exceptionnalisme » pour ouvrir une ère de déstigmatisation, ce qui permettrait d'améliorer la réponse face à l'épidémie dans le monde entier.

## Resumen

Durante la realización de encuestas bioconductuales que miden la prevalencia del contagio del virus de la inmunodeficiencia humana (VIH), los encuestados deberían entregar los resultados de la última prueba de VIH a la que se hayan sometido. No obstante, muchas autoridades gubernamentales, organizaciones no gubernamentales, investigadores y otras partes interesadas de la sociedad civil han declarado que las personas encuestadas implicadas en dichas encuestas no deberían tener que realizar una autodeclaración de su estado con relación al VIH. Las razones expuestas para no pedir a los encuestados que informen de su condición son que las respuestas pueden ser inexactas y que preguntarles por su estado en relación al VIH puede violar sus derechos humanos y exacerbar la estigmatización y la discriminación. Sin embargo, se sostiene que, en la era de terapias antirretrovirales, solicitar a los encuestados en encuestas bioconductuales que declaren su estado en relación al VIH es fundamental para medir y mejorar el acceso a, y la cobertura de, servicios para la atención, el tratamiento y la prevención del contagio del VIH. También es importante para estimar el verdadero alcance de las necesidades insatisfechas a la hora de abordar la epidemia de VIH, así como para interpretar las conductas asociadas a la adquisición y transmisión del virus de forma adecuada. Los datos disponibles indican que la mayor parte de los participantes en encuestas relacionadas con la salud están dispuestos a responder una pregunta sobre su estado en relación al VIH como una de las muchas posibles preguntas delicadas sobre comportamientos sexuales y de consumo de drogas. Por último, normalizar las declaraciones sobre el estado en relación al VIH podría ayudar a la comunidad mundial a pasar de una época caracterizada por el “excepcionalismo” a una caracterizada por la “desestigmatización”, y, de este modo, mejorar la respuesta frente a las epidemias a nivel mundial.

## ملخص

يجب أن يُسأل المشاركون في المسوح السلوكية الحيوية التي تقيس معدل انتشار العدوى بفيروس العوز المناعي البشري (HIV) عن نتائج آخر اختبار خضعوا له للكشف عن الإصابة بالفيروس. إلا أن العديد من السلطات الحكومية والمنظمات غير الحكومية والباحثين وغيرهم من الجهات المعنية في المجتمع المدني قد أعلنوا أن المشاركين في مثل هذه المسوح لا يجب أن يُطلب منهم الإفصاح عن وضعية إصابتهم بفيروس العوز المناعي البشري. وتتمثل الأسباب المقدمة لعدم توجيه الطلب إلى المشاركين للإبلاغ عن حالتهم في شبهة عدم دقة الإجابات، كما أن السؤال عن حالة الإصابة بفيروس العوز المناعي البشري قد ينتهك حقوق الإنسان للمشاركين ويثير في نفوسهم الشعور بالحرج الاجتماعي والتمييز. ومع ذلك، فإننا نؤكد على أنه في أوقات العلاج بمضادات الفيروسات، يكون طلب الإبلاغ ذاتيًا عن حالة الإصابة بفيروس العوز المناعي البشري من المشاركين في المسوح السلوكية الحيوية أمرًا ضروريًا لقياس وتحسين سبل الوصول إلى خدمات الرعاية والعلاج والوقاية من عدوى فيروس العوز المناعي البشري، ومدى التغطية التي تقدمها تلك الخدمات. وهذا الأمر مهم أيضًا للوقوف على الحجم الحقيقي لمقدار العجز في تلبية احتياجات التصدي لوباء فيروس العوز المناعي البشري، والوصول إلى تفسير سليم للسلوكيات المرتبطة بانتقال والإصابة بعدوى فيروس العوز المناعي. تشير البيانات المتاحة إلى أن معظم المشاركين في المسوح المتعلقة بالصحة على استعداد للرد عن أي سؤال يتعلق بحالة إصابتهم بفيروس العوز المناعي البشري – باعتباره سؤالاً من بين عدة أسئلة حساسة محتمل طرحها عن السلوكيات الجنسية وتعاطي المخدرات. وأخيرًا، يمكن أن يسهم تطبيع إجراءات الإبلاغ الذاتي عن حالة الإصابة بفيروس العوز المناعي البشري في مساعدة المجتمع العالمي للمضي قدمًا من عصر ما يسمى "الاستثنائية" إلى عصر "التخلص من الحرج الاجتماعي" – وبذلك يتم تحسين سبل التصدي للوباء في جميع أنحاء العالم.

## 摘要

在开展测量人体免疫缺损病毒 (HIV) 发病率的生物行为调查中，应该询问受访者上次的 HIV 检测结果。 但是，许多政府主管部门、非政府组织、研究人员以及其他民间社会利益相关者表示不应该要求参与此类调查的受访者自我报告其 HIV 状况。 他们对此给出的解释是受访者给出的回答可能不准确，而且询问受访者的 HIV 状况可能侵犯了他们的人权，并加剧其耻辱感和受歧视感。 然而，我们认为在抗逆转录病毒治疗时期，要求受访者在生物行为调查中自我报告其 HIV 状况对衡量和改进 HIV 感染护理、治疗和预防服务以及此类服务的覆盖面至关重要。 此外，在评估在应对 HIV 流行病方面未满足的真正需求量以及正确解释与 HIV 的感染与传播相关的行为方面，自我报告也非常重要。 现有数据表明，大多数健康相关调查的参与者愿意回答与 HIV 状况相关的问题，该问题为可能与性和吸毒行为相关的若干敏感问题之一。 最后，对 HIV 状况的自我报告的标准化有助于国际社会从所谓的例外主义转向一种反污名化态度，从而改善全球范围内对流行病的应对水平。

## Резюме

В рамках биоповеденческих исследований, нацеленных на определение распространенности заражения вирусом иммунодефицита человека (ВИЧ), респондентов следует опрашивать относительно результатов их последнего тестирования на ВИЧ. Однако многие государственные органы, неправительственные организации, исследователи и прочие заинтересованные члены гражданского общества заявляли, что респондентам, участвующим в таких исследованиях, не следует задавать вопросы относительно собственного ВИЧ-статуса. Доводы против просьб о самостоятельном сообщении респондентами своего статуса сводятся к тому, что ответы могут быть неточными и что вопросы о ВИЧ-статусе могут являться нарушением человеческих прав респондентов и усугубить стигматизацию и дискриминацию. Несмотря на это, авторы данной статьи настаивают, что в эпоху антиретровирусной терапии необходимо просить респондентов, участвующих в биоповеденческих исследованиях, самостоятельно сообщить о своем ВИЧ-статусе. С помощью этих сообщений удастся определить и улучшить доступность служб для предоставления людям, зараженным ВИЧ, соответствующей помощи и лечения, для профилактики этой инфекции, а также охват такими службами. Кроме того, это важно для оценки реального объема неудовлетворенных потребностей в борьбе с эпидемией ВИЧ и для правильной интерпретации поведения, сопряженного с заражением и передачей ВИЧ. Судя по имеющимся данным, большинство участников исследований состояния здоровья готовы ответить на вопрос о ВИЧ-статусе, являющийся одним из нескольких возможных вопросов конфиденциального характера, касающихся сексуального поведения и употребления наркотиков. В конечном счете признание самостоятельных сообщений о ВИЧ-статусе нормальным явлением могло бы содействовать переходу мирового сообщества от эпохи так называемой исключительности к эпохе дестигматизации, а также совершенствованию реагирования на эпидемию во всем мире.

## Introduction

Community-based surveys such as population-based household and bio-behavioural surveillance surveys are primarily intended to assess the magnitude of epidemics of human immunodeficiency virus (HIV) infection and to evaluate national responses. These surveys are conducted among general populations and key populations at higher risk of HIV exposure and acquired immunodeficiency syndrome (AIDS) – e.g. men who have sex with men, people who inject drugs, and sex workers and their clients. After a decade of scaling-up antiretroviral therapy (ART) and growing evidence of ART’s preventive effects on onward HIV transmission,^1^ evaluation of national responses requires data on each point in the cascade of engagement in HIV-related care.^2^ Data are also required to determine if countries have achieved – or are likely to achieve – the 90–90–90 targets of the Joint United Nations Programme of HIV/AIDS (UNAIDS). UNAIDS set a goal that, by 2020, 90% of people living with HIV will know their HIV status, 90% of people with diagnosed HIV infection will receive sustained ART and 90% of people receiving ART will have viral suppression.^3^ To inform the cascade of engagement in care and monitor progress towards the 90–90–90 targets, it is essential to assess self-reported HIV status in bio-behavioural surveys. We need to know if respondents in such surveys know their serostatus and are enrolled and retained in HIV-related care and treatment. The failure to ask many survey participants their current HIV status – i.e. the results of their most recent HIV test – limits our ability to monitor the cascade of HIV-related care.^4^^,^^5^

Population-based household and bio-behavioural surveys^6^ of key populations are important for monitoring HIV prevalence and risk behaviours. Data from such surveys can improve the design and evaluation of HIV intervention programmes. If survey participants are offered rapid tests for the detection or confirmation of HIV infection, they can be referred to existing HIV services for the confirmation of infection or directly to HIV prevention, care and treatment services.^7^^,^^8^

The behavioural questionnaires found in most population-based and bio-behavioural surveys on HIV ask sensitive questions on both sexual practices – e.g. on the number of sexual partners and condom use – and behaviours that may be illegal in the study country – e.g. on drug use, selling sex and buying sex. Most such surveys also ask participants a question for UNAIDS Global AIDS Response Progress Reporting: “Have you had an HIV test in the past 12 months and, if so, did you receive the test result?”^4^ However, many survey interviewers do not ask respondents to share the results of any HIV tests they have had and therefore cannot ask about respondents’ engagement in care and treatment services. Surveys on HIV must be approved by national and often international human subjects’ review boards and usually record no personal identifying data. To facilitate analysis of the determinants associated with prevalent HIV infections, any self-reported behavioural risk data and HIV test results that are collected are linked by non-identifying reproducible codes. For bio-behavioural surveys among key populations, community advisory groups may be engaged to assure respect for the community^6^ However, most of these surveys are unable to identify any of the important correlates of having unrecognized HIV infection or to make distinctions between the behaviours of those who are aware of their HIV-positive serostatus and those who are unaware.

Unless we can analyse the factors associated with undiagnosed infection and HIV-related care and treatment uptake, we will fail to understand the potential for the expansion of either the HIV epidemic among a given population or the coverage of related care and treatment services. Early treatment – which is only possible with early diagnosis – improves the health and long-term prognosis of people living with HIV and decreases the risk of onward sexual transmission of the virus.^2^^,^^9^^–^^12^

To determine the HIV serostatus of survey participants accurately and inform progress towards the target of 90% of people living with HIV knowing their status, surveys need to ask each respondent who has been tested for HIV about the result of their most recent HIV test. This information is important for planning targeted interventions – including prevention messaging and strategies – and in the exploration of respondents’ experiences of HIV-related stigma and discrimination. Surveys that record self-reported HIV status should include community consultation processes to inform survey implementation and ensure respect of human rights, confidentiality and redress.

The 2012 Kenya AIDS Indicator Survey^13^ is an example of a population-based household survey in which participants were asked their HIV status – providing the opportunity to describe the community-based cascade of care among people living with HIV. This survey found that, of 363 respondents who claimed to be HIV-positive, 89.3% were enrolled in HIV care programmes, 84.5% of those eligible for treatment were receiving ART and 78.5% of those on ART who had not missed taking a pill were virally suppressed.^13^ These results contributed to reprioritization in the HIV response, with greater focus placed on identifying HIV-infected individuals by expanding all testing modalities – particularly among men and children – with a direct linkage between the point of HIV diagnosis and HIV care services.^14^

Despite precautions taken to protect respondents during population-based household and bio-behavioural surveys, many government authorities, nongovernmental organizations, researchers and other civil society stakeholders currently oppose asking respondents to self-report their HIV status. Guidelines for AIDS indicator and bio-behavioural survey questionnaires do not recommend questions on HIV status.^5^^,^^6^^,^^8^ Resistance to the inclusion of data collection on self-reported HIV status has been based on numerous concerns, including that collection of such data could: (i) violate the respondents’ human rights, by inadvertent release of data; (ii) exacerbate stigma and discrimination; and (iii) lead to inaccurate estimates of HIV prevalence. Below, we explore and respond to each of these concerns and present recommendations on how best to include self-reported HIV status in survey questionnaires.

## Protecting human rights

The response to HIV in the 1980s and 1990s led to the advent of so-called AIDS exceptionalism – in part due to human rights abuses and discrimination against people living with HIV.^15^ The initial responses to AIDS included potential and real violations of privacy – including discussions of compulsory quarantine for those at high risk of HIV infection and isolation for those already infected. While stigma and discrimination remain, the need for an exceptional response as it applies to surveys – i.e. the exclusion of questions on HIV status – has decreased because of the introduction of safeguards to respect individual human rights, the availability of ART and the ability of ART to reduce onward HIV transmission risks and improve health outcomes when initiated early after infection.^9^^,^^16^^–^^18^ By asking respondents to state their HIV status – if known – it becomes possible to check if respondents living with HIV are accessing relevant care and treatment services. Individual autonomy can be maintained because respondents can refuse to answer any survey questions or stop the interview at any time, without repercussions. The study protocols and informed consent procedures and forms used in any survey need to be tailored to the HIV-related legal, policy and social context in which the survey is implemented and to the type of HIV testing and procedures for the return of test results and counselling used in the survey area.

## Stigma and discrimination

Key populations and people living with HIV often face layered stigma, discrimination and – depending on their country of residence – the consequences of legislation outlawing behaviours that may increase their risk of HIV infection – e.g. drug use, sex work, sex with sex workers and male–male sex.^19^ Those who oppose asking survey participants to reveal the result of their most recent HIV test are concerned that respondents will suffer additional stigma and discrimination if their self-reported HIV statuses are inadvertently disclosed. To help ensure the confidentiality of test results and minimize the respondent’s risk of further stigma and discrimination, interviewers are often asked to preface any question about a respondent’s HIV test with the words “I do not want to know the results of the test …” Any post-survey stigma and discrimination must be minimized through confidentiality procedures for the survey participants, ethical reviews and staff training on confidentiality. Clear penalties for the disclosure of information on HIV status must be set and should apply to all staff involved in the survey.

Internalized stigma is more difficult to measure and may worsen for survey respondents asked about their HIV status. Bio-behavioural surveys on HIV infection often include questions that most would consider to be sensitive – e.g. on sexual intercourse, condom use and illicit drug injection. Participants are often asked about being arrested, whether they have been forced to have sex and whether they have been discriminated against because of their behaviours. Interviewer training should address how to respond to sensitivities that can vary among individual respondents – including those who know they are HIV-infected.

A review of results from selected bio-behavioural surveys in which respondents – from different key populations in low- and middle-income settings – were asked to state their HIV status – if known – revealed that the proportion of respondents with an HIV testing history who refused to reveal their HIV status varied greatly (Table 1). In a survey of people who injected drugs in Nador, Morocco, none of 277 respondents refused to self-report their HIV status although 4% refused to answer questions on other sensitive topics, such as needle sharing.^22^ In contrast, 15–16% of 6106 people who injected drugs in Ukrainian cities refused to share their HIV status with interviewers.^25^ In a survey in Ghana, where male–male sex remains illegal, only 2% of 456 interviewed men who had sex with men refused to respond to a question about HIV status.^20^ Data we collected on response rates (Table 1) indicate that, in general, survey respondents do not consider a question on HIV status any more sensitive than several other questions included in bio-behavioural studies on HIV.

**Table 1:** Percentages of participants refusing to respond when asked sensitive questions in bio-behavioural surveys, 2007–2012

Region, country and city	Year	No. of respondents	Population	Refused to answer (% of respondents)	
				Question on HIV status^a^	Other sensitive question^b^
**African**					
Ghana					
Accra/Tema^20^	2011	456	MSM	2.0	0.0
Kenya					
Nairobi^21^	2011	596	FSW	0.9	NR
Nairobi^21^	2011	563	MSM	1.2	NR
National^c^	2007	15 853	General	1.8	NR
National^c^	2012	11 626	General	1.6	NR
Morocco					
Nador^22^	2011	277	PWID	0.0	4.0
Tanger^22^	2011	268	PWID	5.0	7.0
**South-East Asia**					
Thailand					
Bangkok^23^	2007	707	FSW	1.9	1.1
Bangkok^24^	2009	742	PWID	0.2	2.2
Chiang Mai^24^	2009	309	PWID	0.0	0.0
Chiang Rai^23^	2007	366	FSW	4.8	1.6
**European**					
Ukraine					
17 cities^25^	2009	1981	PWID	16.0	5.0
26 cities^25^	2011	4125	PWID	15.0	2.0
**Western Pacific**					
China					
Beijing^26^	2011	500	MSM	0.0	0.0

FSW: female sex workers; HIV: human immunodeficiency virus; MSM: men who have sex with men; NR: not reported; PWID: people who inject drugs.

^a^ Respondents were asked the result of their most recent HIV test.

^b^ People who inject drugs were asked if, in the previous 30 days, they had used a syringe that had been previously used by someone else. Men who have sex with men were asked if they had used a condom during their most recent sexual intercourse with a stranger or a person that they did not know well. Female sex workers were asked if they had any signs or symptoms of a sexually transmitted infection – e.g. genital or anal ulcers or genital discharge – in the previous 12 months.

^c^ Data collated by Andrea A Kim of the United States Centers for Disease Control and Prevention.

## Inaccuracy of self-reported status

Many who believe survey respondents should not be asked to reveal their HIV status also believe that the results of such questioning are likely to be inaccurate because of the effects of social desirability bias and post-test seroconversion. The accuracy of self-reports of the most recent HIV test results depends on numerous factors – e.g. to whom and in which context the information is being reported and the perceived benefit or harm of sharing the information. Inaccuracies may arise when respondents fail to understand the meaning of a positive or negative test result,^27^ fail to remember the result or – perhaps because of low social desirability for frequent testing in a particular community^28^ – have not been tested for years. Detailed analysis of data from household surveys in Malawi^29^ and Uganda^30^ indicated that, even after adjusting for expected seroconversions, about one-quarter to one-third of HIV-positive respondents intentionally misreported their HIV status as negative. Conversely, of 4125 participants who provided their serostatus in a 2011 Ukraine survey of people who inject drugs, 85 (2%) who claimed to be seropositive for HIV were found to be seronegative when tested using a rapid diagnostic assay during the survey.^25^ In the 2007 Kenya AIDS Indicator Survey, 92.2% of the 181 respondents who reported that they were HIV-positive were found to be seropositive when tested in the survey and 93.8% of the 4886 respondents who reported that they were HIV negative were found to be seronegative when tested during the survey (Andrea A Kim, United States Centers for Disease Control and Prevention, unpublished data, 2015).

Knowledge of HIV serostatus is important for gauging the level of status awareness among those infected and uninfected with HIV and permits better assessment of the uptake of appropriate services based on HIV status. Although all so-called sensitive questions are subject to response biases, such biases can be minimized by training interviewers to build rapport, assess responses, probe for more valid answers and behave ethically. In some settings, the impact of such biases can be assessed by testing for antiretroviral drugs in the blood. In the 2012 Kenya AIDS Indicator Survey, testing for such drugs indicated that 10% more respondents were aware that they were HIV-infected than indicated by the self-reporting.^13^ In household surveys in Malawi and Uganda, similar testing for antiretroviral drugs indicated similar levels of misreporting of serostatus.^29^^,^^30^ Such evaluation of bias permits improvement of HIV prevalence estimates and suggests that eliciting honest responses to questions about HIV status requires more work to build trust between interviewees and interviewers.

Population-based household surveys increasingly rely on rapid HIV testing in the household – rather than central laboratory testing – for estimating national and subnational HIV prevalence. Accurate data on reasons for refusing rapid HIV testing – e.g. prior known HIV-positive status – must be collected if HIV prevalence estimates from these surveys are to be accurate and unbiased. If confidentiality can be enhanced – e.g. by not collecting names or other personal identifying information and by allowing respondents to record their own data on laptop or tablet computers – then responses are more likely to be honest (Table 2).

**Table 2:** Recommendations for future bio-behavioural surveys on HIV

Aspect of survey	Recommendations
**Survey design**	
Staffing	Ensure that the appropriate staff are available and trained to ask questions about self-reported HIV status in a professional and confidential manner. Have a fully trained HIV counsellor on the staff. Ensure a psychosocial support counsellor is available if needed.
Survey or interview setting	Ensure that the interview area is safe and allows participants to speak confidentially.
Interviewing technique	Consider using computer-assisted self-interviewing techniques – with audio output and input for illiterate participants – to ensure greater confidentiality and privacy for the participant.
**Questionnaire**	
HIV status question	Typically, ask each participant “Have you ever been tested for HIV?” and, if the participant gives a positive answer to this question, ask “Was your most recent HIV test within the last 6 months, 6–12 months ago or more than 12 months ago?” and “What was the result of your most recent HIV test?” Those who say they have never been tested should be asked “What do you think your HIV status is today?”
Supplementary questions	Participants who report being HIV-positive should be asked “Are you currently enrolled in an HIV care programme? Are you currently taking antiretroviral treatment? If so, did you initiate antiretroviral treatment within the last 6 months or 6–12, 12–24, 24–36, 36–48, 48–60 or more than 60 months ago? Have you had a CD4 count? If so, what was your most recent CD4 count and was it within the last 6 months, 6–12 months ago or more than 12 months ago? Have you had a viral load assay and, if so, what was the result and was the assay within the last 6 months, 6–12 months ago or more than 12 months ago?”
Probe	As all self-report data may at times be inaccurate, it is helpful to have additional questions that may help determine if a response is valid. For example, additional questions about being on HIV treatment or attending specific HIV clinics could help verify or refute a previous self-reported HIV status.
**Ethical considerations**	
Consent	Ensure that all participants undergo an informed consent process that explains the survey objectives, steps, possible benefits and harm of findings. Inform participants of the survey’s confidentiality and data anonymity and of their right to refuse to respond to any questions.
Training	Train staff in the ethical conduct of research, including the maintenance of confidentiality and/or anonymity. Make clear what penalties there are for staff breaking confidentiality. Have all staff sign a confidentiality agreement form.
HIV counsellor	Consider training interviewers in HIV test counselling. An HIV counsellor may be better equipped to provide advice, counselling and referrals to those participants responding that they have positive HIV status.
Referral for care and treatment	Have available information about – and referrals for – local care and treatment.
Data management and confidentiality	Do not collect any personal identification. Link all behavioural data, including self-reported HIV status, using codes. Once completed and reviewed, keep all documents, including those with self-reported HIV status, in a secure and locked location that is only accessible to designated personnel.
**Surveys in small communities**	When surveys take place in small communities, where survey staff might know participants, measures in addition to the ethical considerations mentioned above may be needed. For example, consider having interviewers and other staff recuse themselves if they know the participant. Where possible, bring interviewers and other staff members from other communities and/or use computer-assisted self-interviewing techniques.
**Data use**	
Estimates	Estimate the total size of the epidemic from the numbers of reported and unrecognized cases. Also estimate the percentage of HIV-infected participants who were unaware of their positivity at the time of testing in the survey, and the percentage of HIV-infected individuals who are not receiving care.
Identification	Identify population subgroups and risk behaviours associated with unrecognized or undiagnosed infection to improve the prioritization and targeting of care and treatment services.
Comparison	Compare self-reported HIV status from surveys with any additional and relevant data that are available – e.g. viral load, blood levels of antiretroviral medications and case surveillance data – to inform the interpretation of the true levels of HIV status awareness.

HIV: human immunodeficiency virus.

Formative research is needed to compare self-reported HIV status to assay-determined status and identify determinants of discrepancies – e.g. whether responses were purposely incorrect and, if so, why, whether a respondent knows they are HIV-infected but is not on ART, whether status was not accurately known or whether the experience of responding about HIV status varies according to the respondent’s HIV status. Discrepancy between self-reported HIV status and the respondent’s perceived status has been reported.^28^ While asking for known HIV status remains crucial, the resultant data have to be compared with any additional relevant data so that discrepancies between actual, self-perceived and self-reported HIV status can be evaluated.^31^ A deeper understanding of the context in which HIV status is correctly known and reported can be used to enhance survey and questionnaire design and to improve HIV testing and counselling programmes.

## Need for self-reported HIV status

When institutional review boards approve surveys, they expect adherence to informed consent with appropriate protection of human participants – including protections of anonymity, confidentiality and awareness of the right to refuse to respond.^32^ Surveys can include linkages, where needed, with psychosocial support counsellors. We assert that the benefits of asking respondents to self-report their HIV status outweigh the potential risks of asking this question.

### Treatment and care coverage

Questions about knowledge of status provide an introduction to further questions about linkages to HIV care services and ART. In the era of ART, it is especially crucial to assess the reach and coverage of ART programmes and identify – and refer for care – any seropositive individuals not currently in an HIV care programme. Bio-behavioural surveys have been critical in measuring the coverage of HIV prevention programmes. The omission from such surveys of self-reported HIV status represents a missed opportunity to measure treatment coverage, particularly among key populations. Measuring HIV care cascades – in terms of knowing an individual’s HIV status, assessing seropositive individuals for treatment eligibility, enrolment and retention of eligible individuals in pre-ART or ART-based care, and the achievement of viral suppression – is essential to the accurate monitoring of global efforts to slow the HIV epidemic and increase healthy outcomes for people living with HIV.^33^^–^^35^

### Treatment needs and transmission

HIV case reporting captures the number of diagnosed people living with HIV in a population – when mortality and migration are known. However, to estimate the total number of people living with HIV – i.e. total treatment need – the number of undiagnosed cases of HIV infection is required.^36^ Individuals who are unaware of their HIV infection are more likely to be associated with continued HIV transmission.^37^^–^^41^ Comparison of the characteristics of people aware and unaware of their HIV status can lead to more efficient programme design and implementation. Status awareness is also a key parameter in the modelling of HIV transmission rates.

Unrecognized HIV infections are an important factor in onward transmission of the virus and the number of infected individuals who are unaware of their HIV status is a crucial indicator of potential HIV spread.^42^^,^^43^ Infected individuals who believe themselves to be uninfected may use harm-reduction strategies – e.g. seroadaptive behaviours during unprotected sex – incorrectly.^44^^,^^45^ A growing number of HIV interventions – e.g. early ART for sero-discordant couples and pre-exposure prophylaxis – require awareness of HIV status.^1^^,^^46^ Measures of risk behaviours also increasingly require the context of serostatus – of both the individual of interest and their sex partner(s) – to interpret the potential for transmission. Knowledge of which population segments are most likely to be HIV seropositive – and unaware of it – is useful for prioritizing and tailoring intervention programmes for the people most in need^42^^,^^47^ and for forecasting financial needs for the HIV response. Finally, self-reported HIV statuses can be used to monitor HIV testing uptake and efficiency.^33^^,^^38^^,^^48^

## Recommendations

Table 2 summarizes recommendations for survey designers, data analysts, ethical review committees and civil society. For all surveys, it is essential to receive approval of institutional review boards and to pilot questions to ensure they are designed to respect participants while capturing needed information. Respondents who have never had an HIV test may be asked what they think their HIV status is. Although trained survey staff are usually equipped to ask sensitive questions, in some circumstances it might be better to have an HIV counsellor ask and record questions about HIV status. Surveys in which respondents are asked their HIV status should include strategies for the provision of information on HIV and the referral of seropositive respondents for HIV care and treatment. Follow-up questions and additional analyses are useful for verifying responses and optimizing the usefulness of findings for prioritizing and targeting care and treatment services.

## Conclusion

Surveys of HIV and HIV-related risks should ask for self-reported HIV status. Key indicators for characterizing the HIV epidemic, the reach of care and treatment and the potential for transmission cannot be adequately measured without HIV status or expensive, additional biological testing. The identification and education of individuals who are mistaken about their HIV status should help protect the health of the individuals and their partners and improve national programmes’ ability to provide appropriate services to those most in need. When self-reporting HIV status becomes normalized, with reduced stigma and discrimination, universal access to HIV prevention, care and treatment may be achieved.

## References

[1] Consolidated guidelines on the use of antiretroviral drugs for treating and preventing HIV infection. Geneva: World Health Organization; 2013. Available from: http://apps.who.int/iris/bitstream/10665/128048/1/9789241507431_eng.pdf?ua=1&ua=1 [cited 2015 Dec 26]. 24716260

[2] Gardner EM, McLees MP, Steiner JF, Del Rio C, Burman WJ. The spectrum of engagement in HIV care and its relevance to test-and-treat strategies for prevention of HIV infection. Clin Infect Dis. 2011 3 15;52(6):793–800. 10.1093/cid/ciq243 21367734 PMC3106261

[3] 90-90-90. An ambitious treatment target to help end the AIDS epidemic. Geneva: Joint United Nations Programme on HIV/AIDS; 2014. Available from: http://www.unaids.org/sites/default/files/media_asset/90-90-90_en_0.pdf [cited 2015 Dec 26].

[4] Global AIDS response progress reporting 2014: Construction of core indicators for monitoring the 2011 United Nations Political Declaration on HIV and AIDS. Geneva: Joint United Nations Programme on HIV/AIDS; 2014. Available from: http://www.unaids.org/sites/default/files/media_asset/GARPR_2014_guidelines_en_0.pdf [cited 2015 Mar 19].

[5] Behavioral surveillance surveys (BSS). Guidelines for repeated behavioral surveys in populations at risk of HIV. Arlington: Family Health International; 2000. Available from: http://www.who.int/hiv/strategic/en/bss_fhi2000.pdf?ua=1 [cited 2015 Feb 13].

[6] Guidelines on surveillance among populations most at risk for HIV. Geneva: Joint United Nations Programme on HIV/AIDS; 2011. Available from: http://www.unaids.org/sites/default/files/en/media/unaids/contentassets/documents/epidemiology/2011/20110518_Surveillance_among_most_at_risk.pdf[cited 2015 Dec 26].

[7] Monitoring HIV impact using population-based surveys. Geneva: Joint United Nations Programme on HIV/AIDS; 2015. Available from: http://www.unaids.org/sites/default/files/media_asset/JC2763_PopulationBasedSurveys_en.pdf [cited 2016 Apr 12].

[8] AIDS indicator survey - interviewer’s manual. Calverton: ICF Macro; 2010. Available from: http://dhsprogram.com/pubs/pdf/AISM1/AIS_Interviewer's_Manual_28Dec2010.pdf [cited 2015 Feb 13].

[9] Cohen MS, Chen YQ, McCauley M, Gamble T, Hosseinipour MC, Kumarasamy N, et al.; HPTN 052 Study Team. Prevention of HIV-1 infection with early antiretroviral therapy. N Engl J Med. 2011 Aug 11;365(6):493–505. 10.1056/NEJMoa1105243 21767103 PMC3200068

[10] TEMPRANO ANRS 12136 Study Group. A trial of early antiretrovirals and isoniazid preventive therapy in Africa. N Engl J Med. 2015 8 27;373(9):808–22. 10.1056/NEJMoa1507198 26193126

[11] HPTN 052 HIV prevention study demonstrates sustained benefit of early antiretroviral therapy. Bethesda: HIV Prevention Trials Network; 2015. Available from: http://www.hptn.org/web%20documents/PressReleases/HPTN%20052%20Press%20Release%20_07.15.2015_FINAL.pdf [cited 2016 Apr 11].

[12] INSIGHT START Study Group. Initiation of antiretroviral therapy in early asymptomatic HIV infection. N Engl J Med. 2015 8 27;373(9):795–807. 10.1056/NEJMoa1506816 26192873 PMC4569751

[13] Kenya AIDS Indicator Survey 2012. KAIS. Final report. Nairobi: National AIDS and STI Control Programme; 2012. Available from: http://www.nacc.or.ke/images/documents/KAIS-2012.pdf [cited 2016 Apr 11].

[14] Maina WK, Kim AA, Rutherford GW, Harper M, K’Oyugi BO, Sharif S, et al.; KAIS Study Group. Kenya AIDS Indicator Surveys 2007 and 2012: implications for public health policies for HIV prevention and treatment. J Acquir Immune Defic Syndr. 2014 5 1;66 Suppl 1:S130–7. 10.1097/QAI.0000000000000123 24732817 PMC4784700

[15] Bayer R, Edington C. HIV testing, human rights, and global AIDS policy: exceptionalism and its discontents. J Health Polit Policy Law. 2009 6;34(3):301–23. 10.1215/03616878-2009-002 19451406 PMC2730828

[16] Nolan S, Wood E. End of the debate about antiretroviral treatment initiation. Lancet Infect Dis. 2014 4;14(4):258–9. 10.1016/S1473-3099(13)70329-3 24602843 PMC4084688

[17] Rodger A, Cambiano V, Bruun T. HIV transmission risk through condomless sex if the HIV positive partner is on suppressive ART: PARTNER study. In: 21st Conference on Retroviruses and Opportunistic Infections, Boston, 3–6 March, 2014.

[18] Grinsztejn B, Hosseinipour MC, Ribaudo HJ, Swindells S, Eron J, Chen YQ, et al.; HPTN 052-ACTG Study Team. Effects of early versus delayed initiation of antiretroviral treatment on clinical outcomes of HIV-1 infection: results from the phase 3 HPTN 052 randomised controlled trial. Lancet Infect Dis. 2014 4;14(4):281–90. 10.1016/S1473-3099(13)70692-3 24602844 PMC4144040

[19] Jain A, Nyblade L. Scaling up policies, interventions, and measurement for stigma-free HIV prevention, care and treatment services. Washington: Health Policy Project; 2012. Available from: Available from http://www.healthpolicyproject.com/pubs/66_WorkingPaperStigmaScaleUpMeasurementJuly.pdf[cited 2016 Apr 11].

[20] The Ghana men’s study: integrated biological-behavioural surveillance surveys and population size estimation among men who have sex with men in Ghana. Accra: Ghana AIDS Commission; 2013.

[21] 2010–2011 integrated biological and behavioural surveillance survey among key populations in Nairobi and Kisumu, Kenya. Nairobi: National AIDS and STI Control Programme; 2014.

[22] Toufik A, Johnston LG. HIV integrated behavioural and biological surveillance surveys-Morocco 2011–2012. Injecting drug users in Tanger and Nador. Rabat: Ministry of Health; 2013.

[23] Thailand Ministry of Public Health – US CDC Collaboration. 2007 respondent-driven sampling survey among female sex workers in Bangkok and Chiang Rai. Nonthabhuri: Ministry of Public Health; 2008.

[24] Thailand Ministry of Public Health – US CDC Collaboration. Respondent-driven sampling survey of HIV-related risk behaviour and HIV prevalence in injection drug users in Bangkok and Chiang Mai, Thailand. 2009. Nonthabhuri: Ministry of Public Health; 2010.

[25] Balakiryeva O, Bondar T, Sereda Y, Sazonova Y. Analytical report: behavior monitoring and HIV prevalence among injecting drug users as a component of second generation surveillance. Kiev: International HIV/AIDS Alliance in Ukraine; 2011. Available from: http://www.aidsalliance.org.ua/ru/library/our/2012/me/idu_en_2011.pdf[cited 2015 Dec 26].

[26] Lu H, Han Y, He X, Sun Y, Li G, Li X, et al. Alcohol use and HIV risk taking among Chinese MSM in Beijing. Drug Alcohol Depend. 2013 12 1;133(2):317–23. 10.1016/j.drugalcdep.2013.06.013 23876859

[27] Ongoing formative research: understanding of HIV testing and serostatus awareness questions [NHBS-IDU 3], principal investigator meeting. Atlanta: United States Centers for Disease Control and Prevention; 2012.

[28] Sanchez TH, Kelley CF, Rosenberg E, Luisi N, O’Hara B, Lambert R, et al. Lack of awareness of human immunodeficiency virus (HIV) infection: problems and solutions with self-reported HIV serostatus of men who have sex with men. Open Forum Infect Dis. 2014 9 13;1(2):ofu084. 10.1093/ofid/ofu084 25734150 PMC4281805

[29] Malawi: demographic and health survey 2010. Zomba: National Statistical Office; 2010. Available from: https://dhsprogram.com/pubs/pdf/FR247/FR247.pdf [cited 2015 Dec 26].

[30] Uganda AIDS Indicator Survey 2011. Kampala: Ministry of Health; 2011. Available from: http://health.go.ug/docs/UAIS_2011_KEY_FINDINGS.pdf [cited 2015 Dec 26].

[31] Fishel J, Barrere B, Kishor S. Validity of data on self-reported HIV status and implications for measurement of ARV coverage in Malawi. Calverton: ICF International; 2012. Available from: http://dhsprogram.com/pubs/pdf/WP81/WP81.pdf[cited 2015 Dec 26].

[32] Protection of human subjects: Title 45 code of federal regulations part 46. Washington: United States Depatment of Health and Human Services; 2009. Available from: http://www.hhs.gov/ohrp/policy/ohrpregulations.pdf [cited 2015 Dec 26].

[33] Gardner EM, Young B. The HIV care cascade through time. Lancet Infect Dis. 2014 1;14(1):5–6. 10.1016/S1473-3099(13)70272-X 24076276

[34] Kuo AM, Haukoos JS, Witt MD, Babaie ML, Lewis RJ. Recognition of undiagnosed HIV infection: an evaluation of missed opportunities in a predominantly urban minority population. AIDS Patient Care STDS. 2005 4;19(4):239–46. 10.1016/S1473-3099(13)70272-X 15857195

[35] Cherutich P, Kaiser R, Galbraith J, Williamson J, Shiraishi RW, Ngare C, et al.; KAIS Study Group. Lack of knowledge of HIV status a major barrier to HIV prevention, care and treatment efforts in Kenya: results from a nationally representative study. PLoS One. 2012;7(5):e36797. 10.1371/journal.pone.0036797 22574226 PMC3344943

[36] Raymond HF, Bereknyei S, Berglas N, Hunter J, Ojeda N, McFarland W. Estimating population size, HIV prevalence and HIV incidence among men who have sex with men: a case example of synthesising multiple empirical data sources and methods in San Francisco. Sex Transm Infect. 2013 8;89(5):383–7. 10.1136/sextrans-2012-050675 23620133

[37] Marks G, Crepaz N, Janssen RS. Estimating sexual transmission of HIV from persons aware and unaware that they are infected with the virus in the USA. AIDS. 2006 6 26;20(10):1447–50. 10.1097/01.aids.0000233579.79714.8d 16791020

[38] Hall HI, Song R, Rhodes P, Prejean J, An Q, Lee LM, et al.; HIV Incidence Surveillance Group. Estimation of HIV incidence in the United States. JAMA. 2008 8 6;300(5):520–9. 10.1001/jama.300.5.520 18677024 PMC2919237

[39] Chkhartishvili N, Sharavdze L, Chokoshvili O, DeHovitz JA, del Rio C, Tsertsvadze T. The cascade of care in the Eastern European country of Georgia. HIV Med. 2015 1;16(1):62–6. 10.1111/hiv.12172 24919923 PMC4264988

[40] Kiriazova TK, Postnov OV, Perehinets IB, Neduzhko OO. Association of injecting drug use and late enrolment in HIV medical care in Odessa Region, Ukraine. HIV Med. 2013 10;14 Suppl 3:38–41. 10.1111/hiv.12059 24033902

[41] Fonner VA, Denison J, Kennedy CE, O’Reilly K, Sweat M. Voluntary counseling and testing (VCT) for changing HIV-related risk behavior in developing countries. Cochrane Database Syst Rev. 2012 9 12;9:CD001224. 22972050 10.1002/14651858.CD001224.pub4 PMC3931252

[42] Raymond HF, Bingham T, McFarland W. Locating unrecognized HIV infections among men who have sex with men: San Francisco and Los Angeles. AIDS Educ Prev. 2008 10;20(5):408–19. 10.1521/aeap.2008.20.5.408 18956982

[43] Feldman MB. A critical literature review to identify possible causes of higher rates of HIV infection among young black and Latino men who have sex with men. J Natl Med Assoc. 2010 12;102(12):1206–21. 10.1016/S0027-9684(15)30776-8 21287902

[44] McFarland W, Chen Y-H, Nguyen B, Grasso M, Levine D, Stall R, et al. Behavior, intention or chance? A longitudinal study of HIV seroadaptive behaviors, abstinence and condom use. AIDS Behav. 2012 1;16(1):121–31. 10.1007/s10461-011-9936-8 21644001 PMC3686637

[45] Snowden JM, Raymond HF, McFarland W. Seroadaptive behaviours among men who have sex with men in San Francisco: the situation in 2008. Sex Transm Infect. 2011 3;87(2):162–4. 10.1136/sti.2010.042986 21076140

[46] Guidelines on when to start antiretroviral therapy and on pre-exposure prophylaxis for HIV. Geneva: World Health Organization; 2015. Available from: http://apps.who.int/iris/bitstream/10665/186275/1/9789241509565_eng.pdf [cited 2015 Dec 26]. 26598776

[47] Young SD, Shoptaw S, Weiss RE, Munjas B, Gorbach PM. Predictors of unrecognized HIV infection among poor and ethnic men who have sex with men in Los Angeles. AIDS Behav. 2011 4;15(3):643–9. 10.1007/s10461-009-9653-8 20043200 PMC3029495

[48] Do TD, Chen S, McFarland W, Secura GM, Behel SK, MacKellar DA, et al. HIV testing patterns and unrecognized HIV infection among young Asian and Pacific Islander men who have sex with men in San Francisco. AIDS Educ Prev. 2005 12;17(6):540–54. 10.1521/aeap.2005.17.6.540 16398576

